# The Unfinished Public Health Agenda of Chagas Disease in the Era of Globalization

**DOI:** 10.1371/journal.pntd.0000470

**Published:** 2009-07-07

**Authors:** Carlos Franco-Paredes, Maria Elena Bottazzi, Peter J. Hotez

**Affiliations:** 1 Hospital Infantil de México, Federico Gómez, México, D.F., México; 2 Department of Medicine, Emory University, Atlanta, Georgia, United States of America; 3 Department of Microbiology, Immunology, and Tropical Medicine, The George Washington University, Washington, D.C., United States of America; 4 Sabin Vaccine Institute, Washington, D.C., United States of America

***“It takes all the running you can do, to keep in the same place. If you want to get somewhere else, you must run at least twice as fast as that!”***

**Lewis Carroll's** ***Through the Looking-Glass*** **** [**1**]

Many neglected tropical diseases (NTDs) began millions of years ago as enzoonotic diseases of wild animals that subsequently infected humans, leading in many cases to anthropozoonoses [2]. Because of their impoverished circumstances, most humans infected with NTD agents have few choices but to face daily the intense selective pressures associated with high levels of exposure and transmission that often flourish in these impoverished settings [3]. In this dynamic and relentless evolutionary battle, animal reservoirs, vectors, microbes causing NTDs, and humans are constantly adapting through what it is known as the “Red Queen Effect” from Lewis Carroll's Red Queen character [4],[5]. Typically, the battlegrounds of these molecular and ecological clashes are located in the poorest regions of developing countries where they mostly affect the world's most vulnerable populations [3].

**Figure 1:**
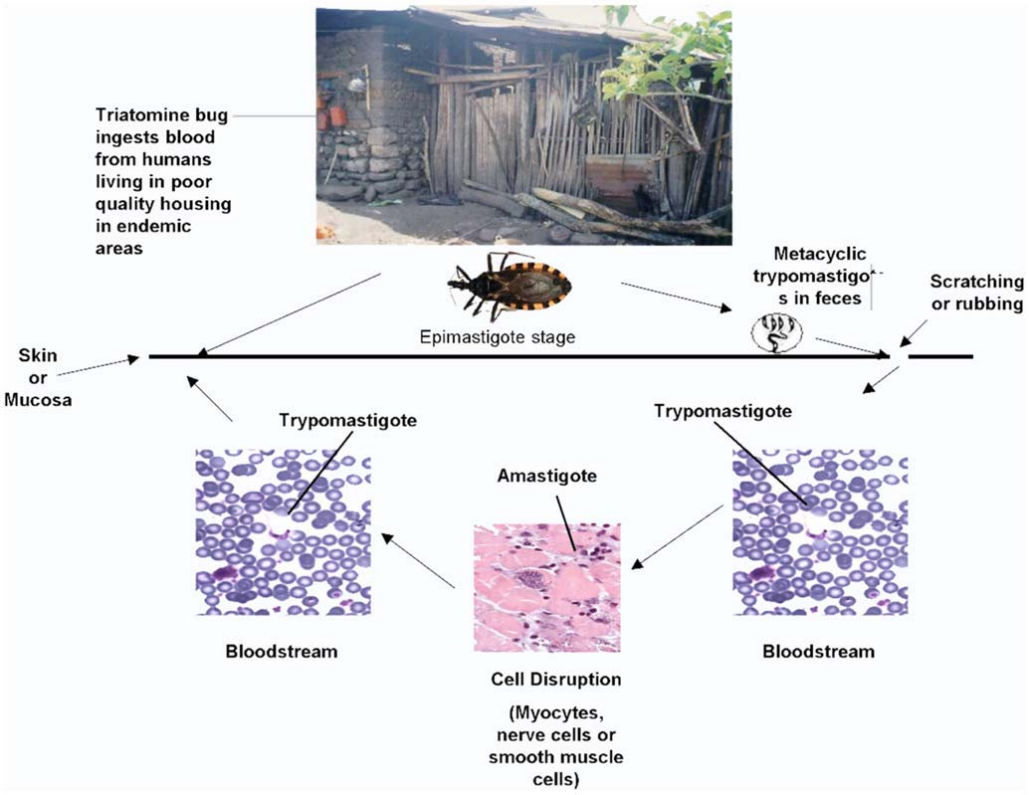
The cycle of transmission of *Trypanosoma cruzi*, the causative agent of Chagas disease to humans. From [3].

In Latin America, Chagas disease (CD), caused by infection with *Trypanosoma cruzi*, is a prime example of this co-evolutionary process in which parasites and mammalian reservoirs (including humans) are engaged in a dynamic race of ecological adaptation and counter-adaptation [6]. In the search for improved sources of income, agriculture, livestock rearing, and other socioeconomic activities, human populations began migrating into the natural wild habitats where *T. cruzi* infection was enzootic [2]. Ultimately, poverty, poor housing and sub-standard living conditions, deforestation, and other ecological factors promoted an adaptation of triatomine vectors to both humans and domestic animals, with increased efficiencies of the wild, domestic, and peridomestic cycles of *T. cruzi* transmission (Figure 1) [2], [7]–[8]. Once humans became infected and acted as a reservoir for the infection, other forms of transmission also evolved through blood transfusion, congenital transmission, and organ transplantation. In some settings, oral transmission through contaminated food is now considered an important mode of transmission [3],[8].

We are about to celebrate the 100th anniversary of the discovery of *T. cruzi* and its link to CD. The cycle of transmission (vector, reservoir, and infectious agent) and the clinical manifestations of the disease were elegantly described by Carlos Chagas (1879–1934) during the first decade of the 20th century. Oswaldo Cruz, his mentor, had set the stage for these achievements by creating a research institution bearing his name in Brazil (Instituto Oswaldo Cruz – FIOCRUZ) [7], [9]–[10]. For his multiple achievements, Carlos Chagas received several awards and international distinctions and became the Director of the Institute in 1917 (after Oswaldo Cruz's death), holding this title until his own death in 1934. It was Salvador Mazza in Argentina who brought again Chagas's achievements into the international scope by identifying cases of CD in the Argentine Chaco region [9]. In subsequent years, a few dedicated clinicians in Latin America expanded the initial clinical descriptions of the disease made by Carlos Chagas. During this period, the French parasitologist Emile Brumpt is often credited with developing important xenodiagnostic techniques [10].

Due to these landmark discoveries, it is now recognized that the natural history of CD has three clinical stages [2],[3]. An initial acute stage representing the entry of the parasite and invasion of the bloodstream in which most patients are asymptomatic is followed by an indeterminate stage that is defined by the absence of symptoms and clinical findings in patients with a positive serology for *T. cruzi*. The indeterminate stage (also called early chronic) is followed by chronic complications in approximately 20%–30% of patients many years after the initial infection. Serious cardiac (e.g., cardiomyopathy) and gastrointestinal (e.g., megaesophagus and megacolon) morbidities are the most frequent manifestations of chronic CD and the main cause of disability and death [2].

During the early 1980s, the prevalence of CD was first reliably estimated and the full social and economic implications of this condition were revealed. It was determined that there were 18 million cases in 21 endemic countries with 100 million people at risk of infection [9]. In 1993, CD ranked as the most important tropical disease in Latin America in terms of burden of disease [9]. Subsequently, through political commitment and effective public health interventions, some progress was achieved in reducing the incidence and prevalence of CD, particularly in the southernmost countries of Latin America. The major approaches to control CD have included improved case management and vector control programs, together with housing improvement through regional programs and blood bank screening [2], [6], [7]–[9]. To date, the major regional initiatives combining some of these interventions have included the Southern Cone Initiative (INCOSUR) created in 1991, followed by the Andean Initiative in 1997 (ACI-IPA), and the Central America and Mexico Initiative (IPCA-MEXICO) and Amazon Initiative (AMCHA) in 2004. As a result of these programs and according to the most recent estimates, the number of people currently infected with CD has been reduced to approximately 7.6 million, with 75 million people still living at risk of infection [11].

**Figure 2:**
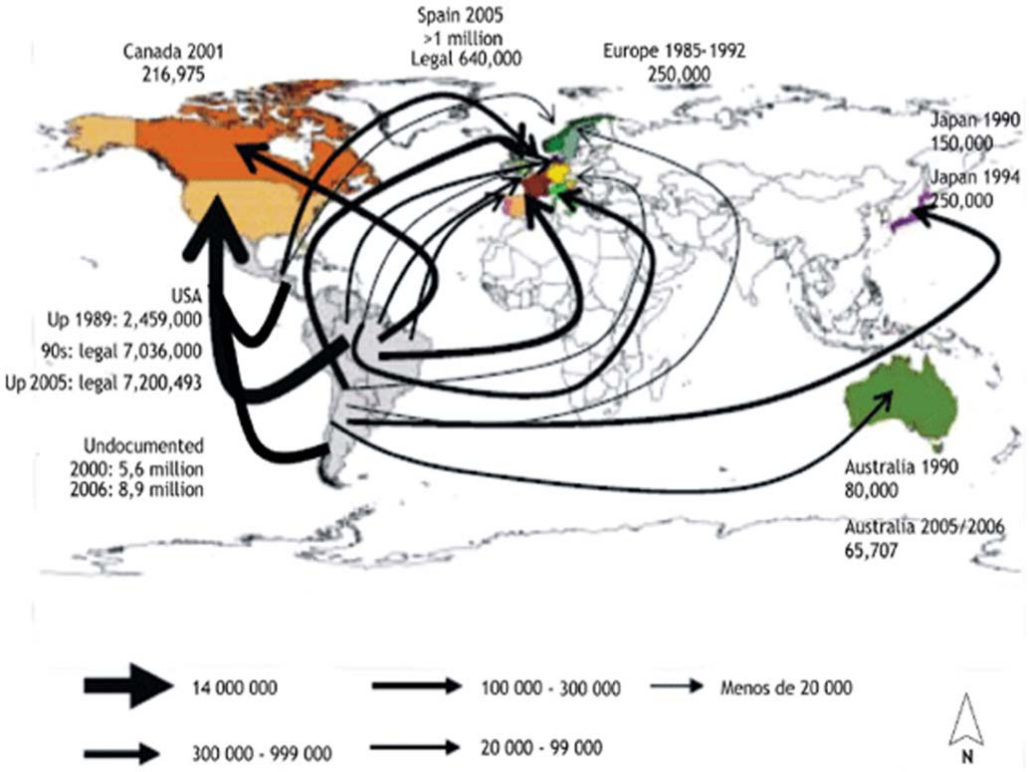
Globalization of Chagas disease from endemic to non-endemic settings. Reprinted with permission from *Memórias do Instituto Oswaldo Cruz* [14].

In spite of these substantial achievements, many challenges remain ahead for the control and ultimately the elimination of CD. Together with the intestinal helminth infections, CD is responsible for the highest estimated disease burden due to infectious diseases in Latin America [6]. In addition, the huge impact of CD on worker productivity and maternal child health continues to contribute to underdevelopment [3],[6]. Some of the successes in the control of the disease are in jeopardy. For instance, in the Southern Cone, where domestic transmission has been nearly eliminated through vector control of *Triatoma infestans*, there are concerns that the vacant niches will be eventually occupied by other triatomine vectors [6],[8]. In other parts of Latin America, poor governance and politically motivated human rights violations have facilitated the emergence and geographic spread of CD [12]. There is even strong evidence for autochthonous transmission within the borders of the United States, especially in the southern states [13]. Additionally, due to regional and global migration caused by economic hardship, the geographic reach of CD has expanded from rural to urban areas in endemic countries as well as in non-endemic countries, a phenomenon sometimes referred to as the globalization of CD (Figure 2) [14].

Current prevention strategies are not enough to control the burden of disease associated with CD. The diagnosis and treatment for every affected individual with CD are moral imperatives and represent a human rights priority [12]. Health care access for diagnosis and treatment is limited in impoverished areas in highly endemic settings [3]. The major antiparasitic drugs used are expensive or toxic, sometimes unavailable, and are not sufficiently effective to treat the chronic forms of the disease [6],[8]. In this and an upcoming issue of *PLoS Neglected Tropical Diseases*, a series of articles coordinated by Médicins Sans Frontiéres (MSF) and Drugs for Neglected Disease Initiative (DNDi) address some of the challenges and ongoing intervention strategies led by these two organizations to ameliorate the medical impact of CD in highly affected areas [15],[16].

In the first report of the series, MSF workers in the field demonstrate from an operational perspective the feasibility of implementing CD diagnosis and treatment protocols in highly affected populations in Honduras, Guatemala, and Bolivia [15]. Through their important contributions in the diagnosis and treatment of CD, MSF has proven the importance of establishing case management protocols that include information, education, and communication at the community and family level, as well as protocols for health staff training with regard to screening, diagnosis, and treatment of CD. This study also confirms the need for better drugs with improved safety profiles, the urgent need for the development of formulations for pediatric cases, and the need for better serological or molecular tests of cure within case management protocols [15]. In another article in the series, Riberio et al. present the current and future state of CD drug development by describing current research and development funding, products in the development pipeline, and products undergoing clinical evaluation [16].

In the coming decade, we anticipate multiple challenges to control or eliminate the medical and public health impact of CD. They include the following:

- scaling of integrated control programs throughout all affected countries by blood bank screening, residual insecticide treatment of vector-infested homes, health promotion and education, and community surveillance of house re-infestation in endemic settings [6];
- improving access to high-quality health care for diagnosis and treatment of CD and its cardiac, gastrointestinal, and neurological complications in endemic settings [15];
- expanding screening, diagnosis, and treatment protocols of CD in non-endemic settings receiving large numbers of migrants from areas of endemicity in Latin America [13],[14];
- expanding screening and treatment to prevent congenital CD transmission in endemic settings [6];
- developing and testing more efficacious and safer drugs for the acute, indeterminate, and chronic forms of the disease [16]; and
- further defining the epidemiologic and clinical manifestations of CD co-infection or reactivation among patients with HIV/AIDS and other immunocompromised populations in endemic and non-endemic settings [17].

In order to address these challenges, and in support of the existing MSF and DNDi initiatives, the Global Network for Neglected Tropical Diseases of the Sabin Vaccine Institute is working in collaboration with the Inter-American Development Bank and the Pan American Health Organization to support large-scale programs for the control of CD and other NTDs in the Latin American region. Simultaneously, several product development partnerships, including DNDi and the Sandler Center at the University of California San Francisco, as well as a new NTD research and development initiative of the Brazilian Ministry of Health, are working to develop new and improved control tools for CD. Once again, FIOCRUZ will be at the forefront of many of these new endeavors.

In summary, there are ongoing social and biological evolutionary races surrounding CD transmission and control on a global scale. We ***must run at least twice as fast*** to increase our efforts to control this poverty-promoting disease. By controlling CD and other NTDs in Latin America, as demonstrated by the programs and strategies of MSF and DNDi, the most vulnerable populations in this region may be in a better position to achieve the Millennium Development Goals.
